# Deep Learning Techniques in the Classification of ECG Signals Using R-Peak Detection Based on the PTB-XL Dataset

**DOI:** 10.3390/s21248174

**Published:** 2021-12-07

**Authors:** Sandra Śmigiel, Krzysztof Pałczyński, Damian Ledziński

**Affiliations:** 1 Faculty of Mechanical Engineering, Bydgoszcz University of Science and Technology, 85-796 Bydgoszcz, Poland; 2 Faculty of Telecommunications, Computer Science and Electrical Engineering, Bydgoszcz University of Science and Technology, 85-796 Bydgoszcz, Poland; krzysztof@palczynski.com.pl (K.P.); damian.ledzinski@pbs.edu.pl (D.L.)

**Keywords:** ECG signal, QRS complex, R wave detection, classification, PTB-XL, deep learning

## Abstract

Deep Neural Networks (DNNs) are state-of-the-art machine learning algorithms, the application of which in electrocardiographic signals is gaining importance. So far, limited studies or optimizations using DNN can be found using ECG databases. To explore and achieve effective ECG recognition, this paper presents a convolutional neural network to perform the encoding of a single QRS complex with the addition of entropy-based features. This study aims to determine what combination of signal information provides the best result for classification purposes. The analyzed information included the raw ECG signal, entropy-based features computed from raw ECG signals, extracted QRS complexes, and entropy-based features computed from extracted QRS complexes. The tests were based on the classification of 2, 5, and 20 classes of heart diseases. The research was carried out on the data contained in a PTB-XL database. An innovative method of extracting QRS complexes based on the aggregation of results from established algorithms for multi-lead signals using the k-mean method, at the same time, was presented. The obtained results prove that adding entropy-based features and extracted QRS complexes to the raw signal is beneficial. Raw signals with entropy-based features but without extracted QRS complexes performed much worse.

## 1. Introduction

The analysis of electrocardiographic signals (ECG) is one of the most important steps in diagnosing cardiac disorders. Research into methods of ECG signal diagnostics has been developed for decades. An electrocardiogram is a commonly employed non-invasive physiological signal used for screening and diagnosing cardiovascular disease. In addition, the signal is used to search for pathological patterns corresponding to diseases. ECG analysis tools require knowledge of the location and morphology of the various segments (P-QRS-T) in the ECG recordings [1]. The most common reference point for assessing ECG signals is the QRS complex and detection of R-waves [2,3,4,5,6]. These studies are complemented by the R–R distance assessment and heart rate analysis as an additional feature of the signal [7,8,9,10,11,12]. It should be noted that these methods usually use databases such as Physionet, PhysioBank, and PhysioToolkit datasets to confirm their performance [13]. Their main goal is to detect arrhythmia—i.e., an abnormal heartbeat—which is a common symptom of heart disease [14].

One of the most common ways that clinicians or cardiologists analyze ECG signals is to inspect these records visually. However, visually assessing ECG signals can be difficult and time-consuming. The authors confirm this in numerous works. Most of these algorithms are based on traditional machine learning and digital signal processing techniques, such as wavelet transform, Fourier Transform, low-pass filters, high-pass filters, median filters, and others [15,16,17,18]. In addition, studies in this area typically involve data preprocessing, feature extraction, and building classifiers using an ECG signal [19,20,21].

Recently, it has been proved that Artificial Intelligence (AI) and Machine Learning (ML) have numerous applications in all engineering fields. The literature includes works in the fields of electrical engineering [22], civil engineering [23], and petroleum engineering [24]. Another group of works includes research based on deep learning (DL) [25,26,27]. The applications of deep learning in biomedical engineering, relevant to this work, have grown exponentially in recent years. Deep learning is the study of information, forecasts, decision making, or the use of a data set, called training data, to identify complex patterns. In particular, DL has been proven to help increase the diagnostic effectiveness of cardiovascular diseases by means of ECG signals. At this time, many researchers have used methods based on deep learning, such as ResNet, InceptionV3, Gated Recurrent Unit (GRU), and Long Short-Term Memory (LSTM) [28,29,30,31]. The learning capabilities of the Convolutional Neural Network (CNN) are successfully employed in ECG signal classification. For example, in one of the studies, DLs were used to detect R waves, reaching an accuracy at the level of 97.22% [32], or for ECG arrhythmia classification, reaching an accuracy at the level of 93.53% [33]. It should be noted here that these tests were conducted on a relatively small number of samples of ECG.

The use of deep learning techniques in ECG signals is a challenge for researchers. A major issue is limited access to the data set. Moreover, deep learning-based methods have high training environments and computing platform requirements, limiting the application scenarios. The solution to the unavailability of data was the PTB-XL database. It is a large, online, publicly available electrocardiography dataset published in April 2020. A large project involving a large number of scientific publications using data from the PTB-XL dataset was PhysioNet/Computing in Cardiology Challenge 2020.

The project PhysioNet/Computing in Cardiology Challenge 2020 was an initiative for authors to research various processes of ECG prediction based on age and gender for the evaluation of signal quality [34,35]. Diverse deep learning models [36] trained on a large dataset of ECG data were used to detect atrial fibrillation. In another study, the authors used the novel convolutional neural network with a non-local convolutional block attention module to solve the problem of detecting arrhythmias in the ECG recording [37]. The classification of cardiac arrhythmias used a deep neural network based on one-dimensional CNN [38], obtaining accuracy results of 0.94–0.97. The authors of [39] undertook the detection and classification of cardiac arrhythmias using long short-term memory. In another study, the authors using DL proposed a Gated Recurrent Unit with the Extreme Learning Machine for ECG signal processing, data sampling, feature extraction, and classification [27]. The authors of [40,41] proposed two SE ResNet models and one rule-based model to improve the classification efficiency of various ECG abnormalities. The use of deep learning techniques in the assessment of the ECG signal was undertaken by the authors of [42], who presented the possibilities of using convolutional neural networks in the classification of heart diseases by examining ECG signals.

This paper presents a comparison of convolutional neural network architectures using different input data combinations. The varieties included the raw ECG signal, entropy-based features computed from raw ECG signals, extracted QRS complexes, and entropy-based features added from extracted QRS complexes. The enrichment of the neural networks with entropy-based features is related to continuing the research [42]. Some works link entropy-based features with biomedical signals in combination with machine learning models. The authors of [43] studied emotional recognition by analyzing the complexity of physiological signals. They assessed the improvement in the efficiency of this process using various characteristics of the entropy domain. They conducted their research based on various physiological signals, including the Electroencephalogram (EEG), Electrocardiogram (ECG), and Galvanic Skin Response (GSR). In their work, they used the XGBoost classifier as a scalable and flexible machine learning method. The authors of [44] conducted similar research, proposing an entropy-based processing scheme for the structure of emotional recognition. The authors’ activities were based on entropy domain feature extraction and prediction by the XGBoost classifier. The analyzed data included EEG, ECG, and GSR signals. The authors used three types of entropy domain features. The proposed scheme for multi-modal analysis outperforms conventional processing approaches. According to the literature review, the area of the usage of entropy-based features as data vectors for machine learning algorithms such as XGBoost is well-established. However, its utilization during Deep Neural Network inference in ECG signal classification is under-researched, and this article aims to explore this set of methods.

The aim of the study was to find the best neural network architectures for disease entities included in 2, 5, and 20 different heart disease classes. In this work, a neural network architecture is defined as a composition of subnetworks called “modules”. Each “module” uses different types of input data: raw signal, extracted QRS complexes, raw signal entropy, and QRS complex entropies. For this purpose, a convolutional neural network was proposed that uses extracted QRS complexes and entropy-based features. In addition, the new method of R-peak labeling and QRS complex extraction has been used. This method uses a 12-lead signal, for which, using the R wave detection algorithms and the k-mean algorithm, the R-peak position estimate is generated. Entropy-based features are promising additions to data preprocessing that may prove beneficial in other signal-processing-related tasks. Examined models are compositions of modules. Each module interprets the different data types, thus creating a heterogeneous architecture instead of typical homogenous neural network structures. Because of that, the proposed architecture has increased computational complexity to obtain better results. Therefore, research on this topic is required.

## 2. Materials and Methods

**Figure 1:**
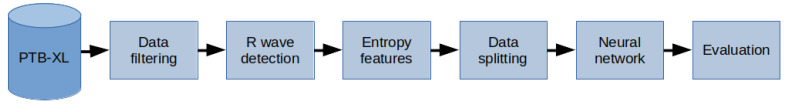
General overview diagram of the method.

The methodology of the research described in this paper is as follows (Figure 1): data from the PTB-XL database were used for the research. The data—i.e., ECG signal records—were filtered. Then, in the raw signal, R-peaks were labeled and split into segments such that there was precisely one ECG R-wave peak in each segment (i.e., QRS complex). Then, the entropy features for the raw signal and the QRS complex were calculated. In the next step, the data were divided into training, validation, and test data, using cross validation. Next, the neural network was trained. The last step was evaluation.

### 2.1. PTB-XL Dataset

In this study, all the ECG data used are derived from the PTB-XL dataset [13,45]. The PTB-XL database is a large dataset containing a set of 21,837 clinical 12-lead ECG records. The sampling rate of the data is 500 Hz and 100 Hz with 16-bit resolution. Each ECG signal is 10 s in length and is annotated by cardiologists. The PTB-XL data are derived from 18,885 patients and are balanced in relation to sex, including 52% of male and 48% of female patients. The dataset involves five major classes: NORM—normal ECG, CD—myocardial infarction, STTC—ST/T change, MI—conduction disturbance, HYP—hypertrophy.

### 2.2. Data Filtering

Initially, the PTB-XL repository contained 21,837 ECG records. However, not all of them are labeled, and not all the labels are assigned 100% certainty. Both cases were filtered out. The remaining records had classes and subclasses assigned to them. In the next step, records with subclasses below 20 were filtered out. This action resulted in the collection of 17,232 ECG records. As a result, each record belonged to one of the 5 classes and one of the 20 subclasses (Table 1). A sampling frequency of 500 Hz was selected for each record of the ECG signal.

### 2.3. R Wave Detection

The P wave, QRS complex, and T wave are the main components in the ECG waveform, of which the QRS complex is its dominant feature. The QRS complex detection is essential in many clinical conditions, including measuring and diagnosing numerous heart abnormalities. The first step in the diagnosis of the QRS complex is R-peak detection.

The PTB-XL databases contain 10 s EGC records. This means that they present records with a constant time but not a constant BPM (beat per minute) number. For this work, these records were cut into sections containing precisely one R wave each.

Determining the R waves from the ECG waveform is not trivial. Therefore, the authors decided to use several detectors. The list of used algorithms is presented below:

- Hamilton detector [46];
- Two average detector [47];
- Stationary Wavelet Transform detector [48];
- Christov detector [49];
- Pan–Tompkins detector [50];
- Engzee detector [51] with modification [52].

**Figure 2:**
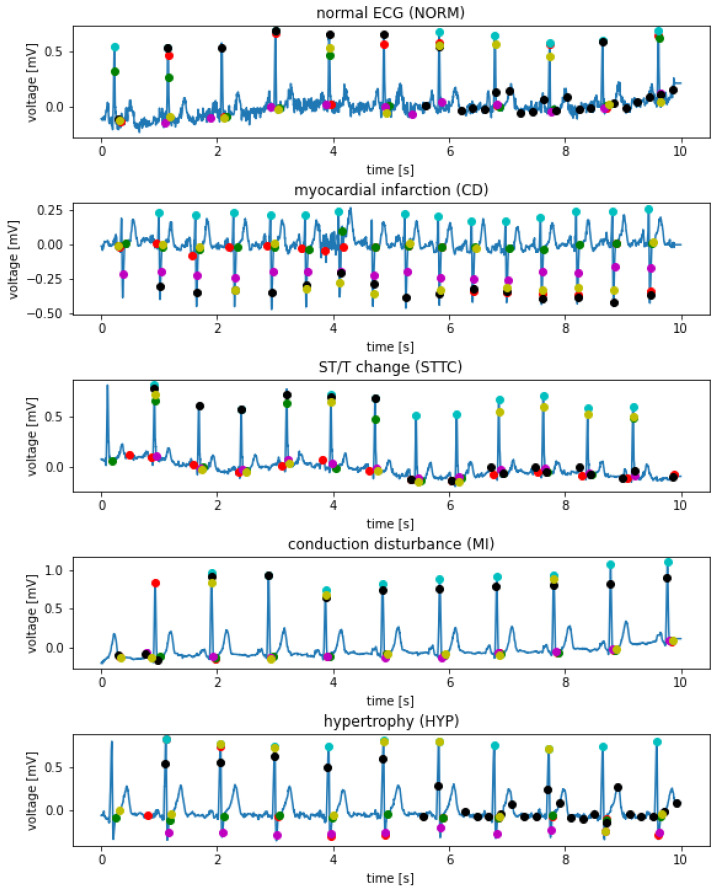
Sample I-lead signals for selected records of various classes with R wave labeled using various techniques.

The methods above return the positions of the R waves in the signal and are designed to work with a single signal (single lead). The PTB-XL database contains 12 lead records. In order to take advantage of the possibilities offered by the base and increase the precision of the algorithm, all 12 signals constituting each record were taken into account. Each of them was processed by all of the detectors. Figure 2 depicts examples of the I-lead signal for selected records of various classes with R waves marked, using various techniques. The following colors are marked accordingly: red—Hamilton detector, green—two average detector, magenta—Stationary Wavelet Transform detector, cyan—Engzee detector, yellow—Pan–Tompkins detector, Black—Christov detector.

In the next step, the computation of the number of R waves in the record was performed. First, the number of R waves from each detector and for each signal (72 in total) was determined. Then, these numbers were used for median calculation. The median is the assumed number of R waves in record $n_{R}$. Hence, the BPM for the record was calculated. The formula describes this process:

(1) $$f_{1},\ldots ,f_{n}:R^{5000}\to \left\{r_{1},\ldots ,r_{n}\right\}$$

(2) $$F=\{f_{i}|i\in N^{+}\cap i\leq n\}$$

(3) $$C_{i}=\left\{\left|F_{j}\left(X_{i}\right)\right|;j\in N^{+}\cap j\leq \left|F\right|\right\}$$

(4) $$\mu_{i,1/2}=C_{i,(\left|C_{i}\right|+1)/2}$$

where $X_{i}$ is the *i*-th ECG signal in the dataset *X*; $f_{1},\ldots ,f_{n}$ are the functions processing signals made of 5000 real-value samples into a set of indexes of R-wave centers; *F* is the set of functions for R-wave extraction; $C_{i}$ is the set of cardinalities of sets of detected R-wave indices extracted by each R-wave detection function for the *i*-th ECG signal; $\mu_{i,1/2}$ is the median of cardinalities of detected R-waves for the *i*-th ECG signal; *n* is the number of functions; and $N^{+}$ are positive natural numbers.

In the next step, a set of points in the one-dimensional space was created, containing the results of all R-wave detectors for all 12 leads to determine the position of the R waves. Then, the application of the k-mean algorithm on the created set was conducted. The number of R-peak $n_{R}$ was assumed as *k*. Finally, the cluster centers of the k-mean algorithm were used to determine the location of the R waves. The evaluation of examined methods was conducted by the computation of the mean absolute error (MAE) of the QRS complex number between the obtained results and ground truth.

**Figure 3:**
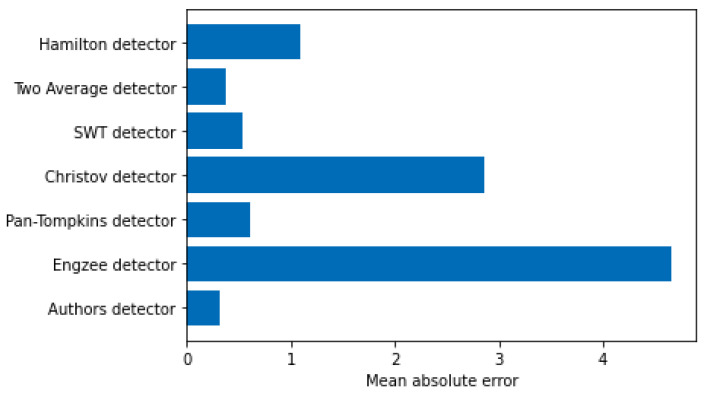
Mean absolute error of the determination of the R-peak number.

**Figure 4:**
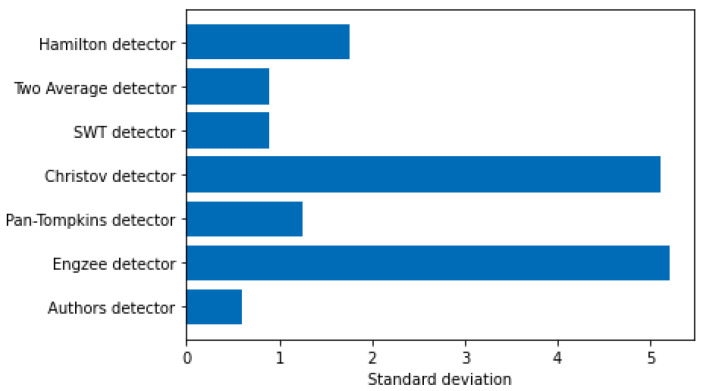
The standard deviation of error of the determination of the R-peak number.

Figure 3 and Figure 4 show a comparison of errors in determining the R-peak number by known detectors and the authors’ detector.

**Figure 5:**
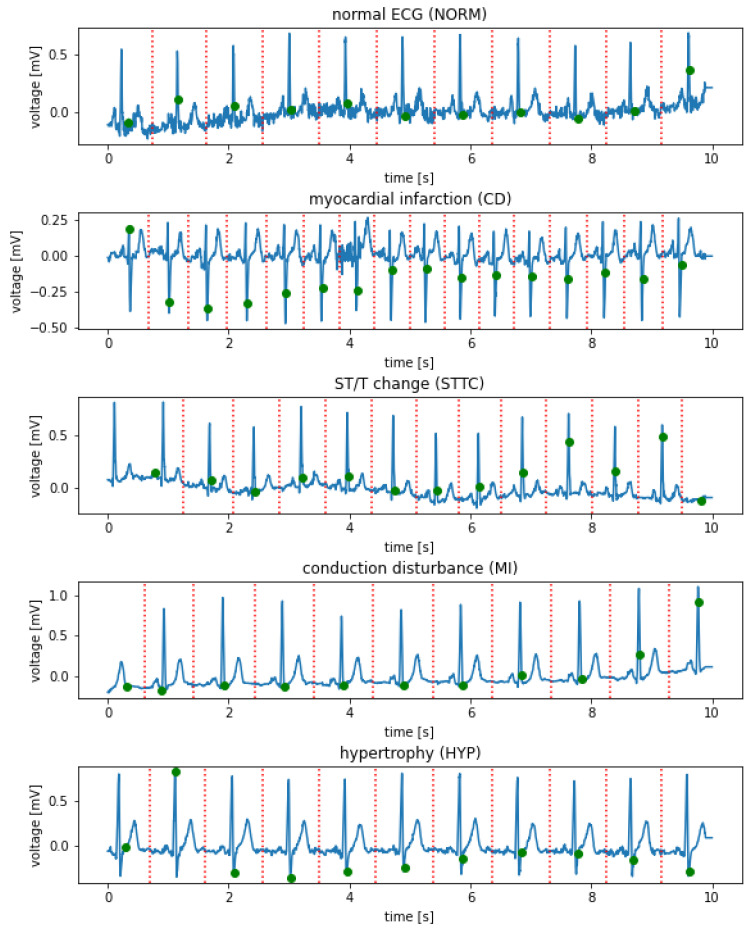
Examples of I-lead signals for selected records of various classes with labeled R wave (green) and places for section cuts (red).

In the next step, a 10 s record was cut with separation points aligned halfway between the R-waves. Finally, the first and last segment were removed. This caused the R wave and QRS to be in the labeled center of the excised section. Figure 5 shows examples of the I-lead signal for selected records of various classes with designated R waves and points of signal cuts.

In the last step, all sections were resampled to obtain 100 measurements per signal. The resampling ratio was kept for each section forming with BPM constituted additional metadata.

### 2.4. Entropy-Based Features

The combination of a neural network with entropy-based features has recently been realized in [42]. In this work, the authors proved that adding entropy-based features to the convolutional neural network ensures the highest accuracy in every classification task. This article examined the utility of measuring ECG and QRS complex information entropies as a feature vector by the deep learning modules specially designed for this task. The entropies listed below have been computed for both raw ECG signals and each individual QRS complex:

- Shannon entropy—quantity of informativeness of signals values [53];
- Approximate entropy—description of how regular values in the time series are and the degree to which the signal is predictable [54];
- Sample entropy—improved version of approximate entropy by disregarding signal size during calculations [54];
- Permutation entropy—quantity describing how deterministic and self-repeating the signal is [55];
- Spectral entropy—description of how uniform the spread of the energy in the frequency spectrum is [56];
- SVD entropy—quantity of possible dimensionality reduction using factorization methods;
- Rényi entropy—more general version of Shannon entropy due to the application of the fractal analysis of the signal [57];
- Tsallis entropy—measurement of long-term memory of the signal and magnitude of its impact on the current values of the signal [58];
- Extropy—quantity of how much uncertainty is associated with the distribution of levels of the signal [59].

According to Granelo-Belinchon et al. [60], information theory measurements can be straightforwardly used in nonstationary signals as long as short periods are considered during which the signal has not changed its parameters yet. Although ECG signals are not stationary, research conducted on the PTB-XL dataset proved that 10 s measurements of heartbeat provide signals that in 89.5% of cases were classified as stationary by the augmented Dickey–Fuller test [61], making these signals stationary with regard to these 10 s long time spans.

### 2.5. Data Splitting

The following data were obtained for each record:

- Raw signal for 12 leads;
- Entropies for raw signal;
- QRS for 12 leads;
- Entropies for QRS;
- Class of record;
- Subclass for record.

Records were divided into training, validation, and test data at the ratios of 70%, 15%, and 15%. To improve the quality of the research, non-exhaustive cross validation was used. For this purpose, the split function was called with five different seed values. This means that all tests were repeated five times for different data splits.

### 2.6. Designed Network Architectures

**Figure 6:**
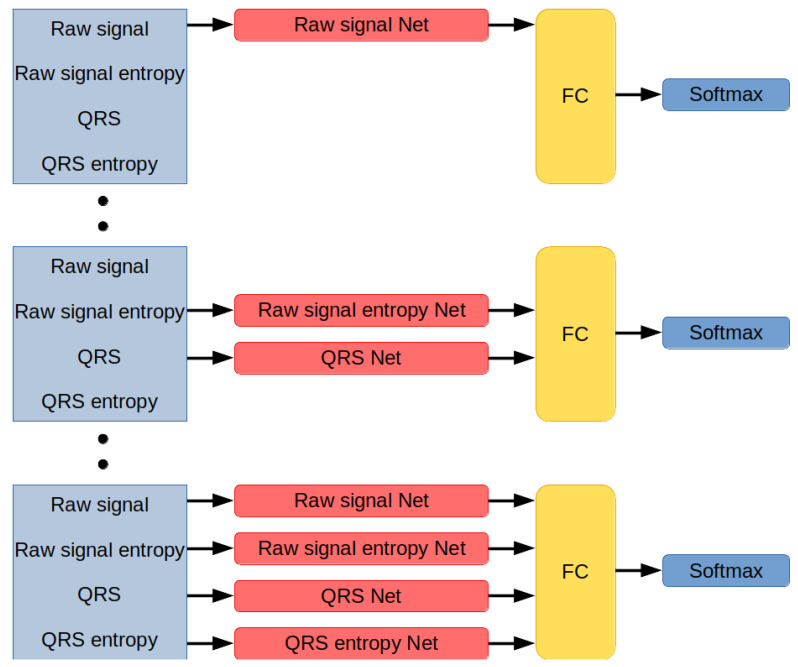
Neural network architectures. Networks are composed of unique combinations of modules, each interpreting a different type of data. The modules’ results are concatenated and processed by a fully-connected layer and softmax function.

Networks developed for this research are modules designed to interpret different types of data (Figure 6). Each module works in parallel with other modules and encodes incoming information into the 20-dimensional vector. The network distributes data among the modules, concatenates their outputs, applies non-linearity by using the Leaky ReLU activation function, inputs them on a fully-connected layer with a number of neurons equal to the number of classes in the classification set, and returns the index of the label associated with the signal.

#### 2.6.1. Module Interpreting Raw Signal

This subnetwork encodes a raw signal. Its input signal contains 5000 samples in each of its 12 channels. The architecture is described in Table 2. A leaky ReLU activation function with a negative slope coefficient of 0.01 was used to process the output of every convolutional layer.

The result of the last convolutional layer is flattened to the 40-dimensional vector and processed by the fully-connected layer with 20 neurons. As a result, the output of this module is a 20-dimensional vector.

The last convolutional layer has a kernel of size 1. Its purpose is to perform the dimensionality reduction of map activation to reduce the number of connections in the fully-connected layer. Without dimensionality reduction, the flatten vector would contain 1920 samples, requiring a fully-connected layer with 38,400 weights to process the output. However, due to applied convolution, the final fully-connected layer has only 800 weights. Thus, in addition to 192 weights required to operate an additional convolutional layer, more than 38 times fewer weights were required to perform the last encoding step.

The architecture of this module is simple yet efficient.

#### 2.6.2. Module Interpreting Entropy-Based Features Calculated for a Raw Signal

This subnetwork encodes vectors of entropy-based features calculated for a raw signal. ECG signal contains 12 channels, and for every channel, 13 entropy-based features have been computed, resulting in a 156-dimensional vector. The architecture is described in Table 3.

#### 2.6.3. Module Interpreting QRS Complex from ECG Signals

This subnetwork processes QRS complexes, aggregating the results and encoding these to the 20-dimensional vector. Each QRS is a 12-channel signal containing 100 samples, but the amount of QRS is not fixed.

**Figure 7:**
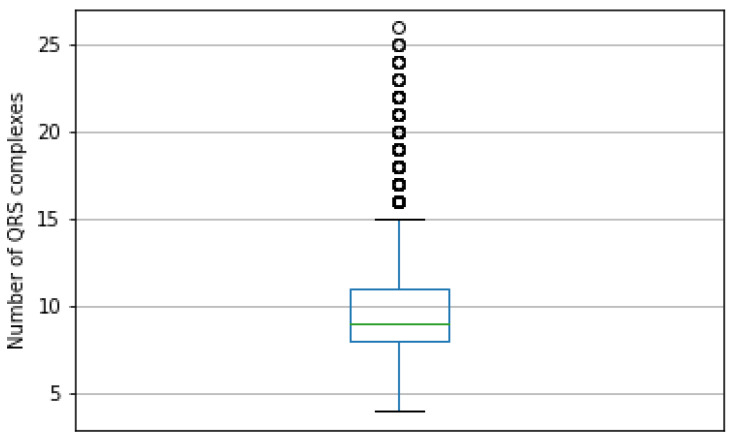
Boxplot presenting distribution of count of QRS complexes in ECG signals of the PTB-XL dataset. The most frequent value is 8, the smallest is 4, and the highest is 15. The outliers are numbers from 16 to 26.

The PTB-XL database contains ECG signals made up of from 4 to 26 QRS signals. The most frequent value of QRS in the ECG signal is 8, with 19.8% occurrence frequency in the dataset. The box plot in Figure 7 presents the distribution of the QRS count in signals.

The number of QRS complexes in the signal has a significant variance, discouraging the solution of this problem by taking the smallest number of signals due to information loss. To handle varying numbers of QRS complexes, the subnetwork is further divided into submodules:

- Single QRS complex encoding function;
- Adaptive Maximum Pooling;
- Adaptive Average Pooling;
- Fully-Connected layer finalizing the computations.

Assume the input data as a set of QRS signals:

(5) $$X_{i}=\left\{QRS_{1},QRS_{2},\ldots ,QRS_{n}\right\};n\in N^{+}$$

We define a wave-encoding function that takes one QRS 12-channel signal containing 100 samples and outputs one 24-dimensional vector:

(6) $$g:R^{12\times 100}\to R^{24}$$

The function is used to encode each QRS in input data:

(7) $$Z_{i}=\left\{g\left(X_{i,j}\right)|j\in N^{+}\cap j<\left|X_{i}\right|\right\}$$

As a result, $Z_{i}$ is a variable-length set of 24-dimensional vectors. This set is now processed by Adaptive Maximum Pooling and Adaptive Average Pooling functions. The Adaptive Maximum Pooling function selects a maximum value for every dimension from vectors in the set:

(8) $$Zmax_{i}=\left[max\left(\left\{Z_{i,j,1}|j\in N^{+}\cap j<\left|X_{i}\right|\right\}\right),\ldots ,max\left(\left\{Z_{i,j,24}|j\in N^{+}\cap j<\left|X_{i}\right|\right\}\right)\right]$$

Adaptive Average Pooling function averages values of every dimension from vectors in the set:

(9) $$Zavg_{i}=\left[\frac{1}{\left|Z_{i}\right|}\sum_{j=1}^{\left|Z_{i}\right|}Z_{i,j,1},\ldots ,\frac{1}{\left|Z_{i}\right|}\sum_{j=1}^{\left|Z_{i}\right|}Z_{i,j,24}\right]$$

The results of both Adaptive Maximum Pooling and Adaptive Average Pooling are concatenated into one 48-dimensional vector:

(10) $$Zall=[Zmax_{i},Zavg_{i}]$$

In the last step, the result is inputted to a fully-connected layer with 20 neurons turning the 48-dimensional vector of concatenated pooling results into a 20-dimensional final vector:

(11) $$Zfinall_{i}=f\left(Zall\right);f:R^{48}\to R^{20}$$

The function performing the encoding of a single QRS complex is performed by a convolutional neural network of the architecture described in Table 4. The leaky ReLU activation function with a negative slope coefficient α of 0.01 was used to process the output of every convolutional layer. The output of the last convolutional layer is flattened to the form of a 24-dimensional layer.

#### 2.6.4. Module Interpreting Entropy-Based Features of Every QRS Signal

This submodule encodes information from entropy-based feature vectors computed for every QRS complex. Due to the varying amount of QRS in the ECG signal, the number of entropy-based feature vectors is also unknown. A neural network set of 156-dimensional feature vectors is aggregated using Adaptive Maximum Pooling and Adaptive Average Pooling functions to adjust input data to fixed-size. Each of these functions generates one 156-dimensional vector. Then, these two vectors are concatenated into one 312-dimensional vector, which is then fed to a shallow neural network. The result is a 20-dimensional vector encoding input data.

The architecture of the neural network is described in Table 5.

### 2.7. Training

Neural networks are trained using the Adam optimizer [62]. Each network is optimized on a train dataset and evaluated on a validation dataset. Training lasts for 10,000 epochs unless early stopping [63] is called. If a network does not improve its best result on the validation dataset in 250 epochs, then training is stopped, and another network is created. The learning rate at the beginning is equal to 0.001, and it is reduced by half if the network does not improve its best result on the training dataset within 50 epochs from the last improvement or learning rate reduction. If the learning rate reaches 0.000001, then no further reduction is applied.

Every epoch consists of 10 batches. Therefore, the batch size is equal to 256. Due to the technical restrictions on the size of Tensors used for GPU computation in PyTorch [64], batch tensors must be made from same-dimensional data. Therefore, only signals of the same number of QRS complexes can be put into the same batch. Because of that limitation, a particular procedure for creating batch tensors was applied.

Preparation phase:

1. Evaluate the data;
2. Find unique numbers of QRS complexes in the dataset;
3. Determine the distribution of QRS complexes numbers in the dataset;
4. Divide set into chunks of data with the same number of QRS complexes.

Batch creation phase:

1. Randomize number of QRS complexes based on distribution established in preparation phase;
2. Select chunk of data based on result of previous operation;
3. If chunk contains less than 256 samples:
  (a) Create tensor from whole chunk;
  (b) Return tensor.
4. If chunk contains more than 256 samples:
  (a) Create tensor from randomly select 256 samples;
  (b) Return tensor.

The training was conducted using hardware configurations on a dual-Intel Xeon Silver 4210R with 192 GB RAM and a Nvidia Tesla A100 GPU. In this research, PyTorch, Sklearn, Numpy, Pandas, and Jupyter Lab programming solutions were used to implement the neural networks [42].

### 2.8. Metrics

Neural networks were evaluated using the metrics described below. For the simplicity of equations, specific acronyms have been created, as follows: TP—true positive, TN—true negative, FP—false positive, FN—false negative. Metrics used for network evaluation are as follows:

- Accuracy: Acc=(TP+TN)/(TP+FP+TN+FN);
- Precision=TP/(TP+FP);
- Recall=TP/(TP+FN);
- F1=2∗Precision∗Recall/(Precision+Recall);
- AUC: Area under ROC. ROC (Receiver Operating Characteristic) is a curve determined by calculating the true positive rate = TFP=TP/(TP+FN) and false positive rate = FPR=FP/(TN+FP). The false positive rate describes the x-axis and the true positive rate the y-axis of a coordinate system. By changing the threshold value responsible for the classification of an example as belonging to either the positive or negative class, pairs of TFP–FPR are generated, resulting in the creation of the ROC curve. AUC is a measurement of the area below the ROC curve.

## 3. Results

To evaluate networks in a way that minimizes the influence of random dataset division, we generated train, validation, and test sets five times. For every module arrangement, a class count and dataset version neural network were created. Each network was trained on a training dataset. During the training, the network was evaluated on the validation dataset to select the best, least overfitted weights set of the network and perform early stopping. When such a set of weights was established, the final network’s evaluation was performed on the test dataset. Results of the networks have been grouped by both modules selection and number of classes. The results are presented in Table 6, Table 7 and Table 8. The tables present the ranges, average value and standard deviation of accuracy, F1 score, and AUC score.

## 4. Discussion

Based on the results, the best model proposed in this article is the composition of modules responsible for interpreting raw signals, QRS complexes, and entropies computed for each QRS wave. This network obtained the best average accuracy on 20 classes, and in other tasks, the accuracy was only around 0.2% on average worse than the best model. The difference is smaller than the standard deviation of the evaluated models. This configuration of modules proved to be the most versatile, scoring an accuracy on average of 90.0% ± 0.4% on 2 classes, 76.2% ± 1.8% on 5 classes, and 68.5% ± 1.3% on 20 classes.

The results prove that adding entropy-based features and extracted QRS complexes to the raw signal is beneficial. In every task, the hybrid network performed the best. The difference between the interpretation of raw signals and other feature supplementation was the highest for predicting 20 classes. The addition of entropy-based features and QRS complexes improved accuracy on average by 6.3%.

Although modules interpreting entropy-based features proved to be, on average, the least accurate models, it is worth noting that these modules were also the simplest, consisting of merely two fully-connected layers. The simplicity of these modules caused by their minimal architecture consisting of only two layers makes their performance impressive, especially for two classes, where QRS entropy achieved on average 86.5% accuracy. Combining this with the fact that the best network in every task used entropy-based features suggests an informational benefit of these metrics.

Their complementation with the base signal may be caused by their different approach to signal interpretation. Convolutional neural networks are designed to extract the information encoded in values of signal samples, their relationship with each other, and the overall shape of the signal. However, entropies are measures of signal predictability, order, and how deterministic they are. These are different ways of extracting information, making them a proper supplementation for signal processing neural networks. The authors plan further research of this phenomenon on other signals.

The entropy-based features extracted from QRS complexes turned out to be better at encoding class-specific information compared to entropy measures of the raw signal. This is a surprising observation. The authors speculated a priori that these entropy-based features would have been less significant than entropy measures conducted on the raw signal due to the structural self-repetitiveness caused by QRS complexes.

R-peak detectors vary in their effectiveness. However, the proposed method of aggregating their results and cross-validating them across signals from several leads simultaneously significantly improves the precision of R-peak detection. In the extracted QRS, the R-peak is not always aligned in the center of the signal’s subsection, which was the authors’ initial goal. This is because the R waves are not at constant distances and the fact that the position of the R wave is determined globally for all 12 leads, which means that for specific leads, especially the extreme ones, a shift may occur.

The methods employed in this research for entropy-based feature calculations and R wave detection have limitations of use due to their computational complexity and non-vectorized code. Therefore, the authors plan to research this subject further to minimize unnecessary computations and vectorize the code, allowing it to use highly optimized computation frameworks such as PyTorch.

The artificial intelligence systems investigated in this article may benefit from feature selection. This procedure may reduce the computational complexity of the networks by calculating only selected entropy metrics (in both raw entropy and QRS complex entropy modules). For example, in [65], the authors applied the Feature Correlation technique to determine useful features in input data. This technique may reduce the amount of required entropy features computation with minimal loss inaccuracy. The authors plan further research on this topic.

## 5. Conclusions

Electrocardiography as a diagnostic tool for detecting heart disease is increasingly supported by algorithms based on machine learning. However, current medical advances are hampered by the lack of appropriate datasets. The answer to these limitations is the PTB-XL database, proposed in work in conjunction with deep learning. The paper presents the use of PTB-XL in the operation of a convolutional neural network, which uses distinguished QRS complexes and entropy-based features. In addition, known algorithms for R-peak detection were tested, and a new detection method was proposed. The conducted tests indicate that single R wave detectors are imperfect, and the presented method allows results to be obtained that are close to the truth. The experimental results for the convolutional neural network showed that the proposed method is reliable and efficient for ECG classification. Furthermore, it was proved that the isolated QRS complexes with entropy-based features significantly improved the results of the operation. Entropy, although it is a general-purpose metric, has proved to be surprisingly effective. The entropy-based features extracted from QRS complexes turned out to be better at encoding class-specific information compared to entropy measures of the raw signal. Undoubtedly, by testing any model on a data set as diverse in terms of diagnostic classes as PTB-XL with a large amount of metadata, it is possible to obtain reliable measurements of the performance of the proposed models. This suggests that deep learning methods could benefit future work on electrocardiographic signals.

## Figures and Tables

**Table 1:** List of classes and subclasses of used records.

Class	Subclass	Number of Records	Description
NORM	NORM	7185	Normal ECG
CD	LAFB/LPFB	881	Left anterior fascicular block, left posterior fascicular block
	IRBBB	798	Incomplete right bundle branch block
	CLBBB	527	(Complete) left bundle branch block
	CRBBB	385	(Complete) right bundle branch block
	IVCD	326	Nonspecific intraventricular conduction disturbance
	_AVB	204	First-degree AV block, second-degree AV block, third-degree AV block
	WPW	67	Wolff–Parkinson–White syndrome
	ILBBB	44	Incomplete left bundle branch block
STTC	STTC	1713	Non-diagnostic T abnormalities, suggests digitalis effect, long QT interval, ST-T changes compatible with ventricular aneurysm, compatible with electrolyte abnormalities
	NST_	478	Nonspecific ST changes
	ISCA	429	In anterolateral leads, in anteroseptal leads, in lateral leads, in anterior leads
	ISC_	297	Ischemic ST-T changes
	ISCI	147	In inferior leads, in inferolateral leads
MI	AMI	1636	Anterior myocardial infarction, anterolateral myocardial infarction, in anteroseptal leads, in anterolateral leads, in lateral leads
	IMI	1272	Inferior myocardial infarction, inferolateral myocardial infarction, inferoposterolateral myocardial infarction, inferoposterior myocardial infarction, in inferior leads, in inferolateral leads
	LMI	28	Lateral myocardial infarction
HYP	LVH	733	Left ventricular hypertrophy
	LAO/LAE	49	Left atrial overload/enlargement
	RAO/RAE	33	Right atrial overload/enlargement

**Table 2:** The architecture of neural network encoding raw signal.

Layer	Channels In	Channels Out	Kernel Size	Padding	Stride
Conv1d	12	24	3	1	1
MaxPool1d	24	24	3	0	3
Conv1d	24	48	3	1	1
MaxPool1d	48	48	3	0	3
Conv1d	48	64	3	1	1
MaxPool1d	64	64	3	0	3
Conv1d	64	72	3	1	1
MaxPool1d	72	72	3	0	3
Conv1d	72	96	3	1	1
MaxPool1d	96	96	3	0	3
Conv1d	96	2	1	0	1

**Table 3:** The architecture of neural network encoding entropy-based features extracted from raw signal.

Layer	Input	Output
Fully-Connected	156	20
Leaky ReLU	20	20
Fully-Connected	20	20

**Table 4:** The architecture of the QRS complex encoding Deep Neural Network.

Layer	Channels In	Channels Out	Kernel Size	Padding	Stride
Conv1d	12	24	3	1	1
MaxPool1d	24	24	2	0	2
Conv1d	24	48	3	0	1
MaxPool1d	48	48	2	0	2
Conv1d	48	96	3	0	1
MaxPool1d	96	96	2	0	2
Conv1d	96	2	1	0	1

**Table 5:** The architecture of neural network encoding entropy-based features extracted from the QRS complexes.

Layer	Input	Output
Fully-Connected	312	20
Leaky ReLU	20	20
Fully-Connected	20	20

**Table 6:** Results for two-class classification.

Name	Acc	Acc Avg|Std	F1	F1 Avg|Std	AUC	AUC Avg|Std
QRS entropy, Raw signal, Raw signal entropy	90.9–89.6%	90.2%|0.5	90.7–89.3	90.0|0.5	96.6–95.5	96.3|0.4
Raw signal, Raw signal entropy	90.7–88.8%	89.8%|0.7	90.6–88.6	89.6|0.7	96.6–95.7	96.1|0.4
QRS entropy, Raw signal	90.6–89.2%	89.9%|0.5	90.4–89.1	89.7|0.5	96.6–95.4	96.1|0.5
QRS, QRS entropy, Raw signal	90.5–89.3%	90.0%|0.4	90.4–89.1	89.7|0.4	96.5–95.1	96.0|0.5
Raw signal	90.5–89.3%	90.0%|0.4	90.2–89.0	89.7|0.4	96.6–95.7	96.2|0.4
QRS	90.5–89.2%	89.7%|0.4	90.2–89.0	89.4|0.4	95.9–94.8	95.5|0.4
QRS, Raw signal entropy	90.5–88.2%	89.8%|0.9	90.3–88.0	89.5|0.9	96.2–95.0	95.7|0.4
QRS, QRS entropy, Raw signal entropy	90.2–89.0%	89.8%|0.4	89.9–88.8	89.5|0.4	96.3–95.8	96.0|0.2
QRS, QRS entropy, Raw signal, Raw signal entropy	90.2–89.3%	89.8%|0.4	90.1–89.0	89.6|0.4	96.5–95.6	96.0|0.4
QRS, Raw signal	90.2–88.7%	89.8%|0.6	90.1–88.6	89.5|0.6	96.4–95.6	96.0|0.3
QRS, QRS entropy	90.1–89.2%	89.7%|0.3	89.9–89.0	89.4|0.3	96.2–95.2	95.9|0.4
QRS, Raw signal, Raw signal entropy	89.9–88.9%	89.5%|0.4	89.6–88.7	89.3|0.3	96.5–95.1	95.9|0.6
QRS entropy	87.0–86.3%	86.5%|0.3	86.7–85.9	86.2|0.3	94.2–92.8	93.6|0.5
QRS entropy, Raw signal entropy	86.7–86.2%	86.6%|0.2	86.4–86.0	86.3|0.2	94.2–93.5	93.8|0.3
Raw signal entropy	83.9–82.1%	83.4%|0.7	83.5–81.6	82.9|0.7	91.6–90.2	91.1|0.5

**Table 7:** Results for five-class classification.

Name	Acc	Acc Avg|Std	F1	F1 Avg|Std	AUC	AUC Avg|Std
QRS, Raw signal, Raw signal entropy	79.1–74.9%	76.3%|1.6	72.0–65.8	68.3|2.4	91.8–88.6	90.3|1.2
QRS, QRS entropy	78.0–75.2%	76.2%|1.0	70.0–66.9	68.0|1.2	91.0–89.4	90.3|0.6
QRS, QRS entropy, Raw signal	77.7–73.6%	76.2%|1.8	70.3–62.7	67.5|3.3	91.8–89.5	90.4|0.9
QRS, QRS entropy, Raw signal entropy	77.4–75.2%	76.0%|0.8	69.6–66.7	68.2|1.3	91.3–90.4	90.7|0.3
QRS entropy, Raw signal, Raw signal entropy	77.2–75.3%	75.9%|0.7	70.5–66.5	67.7|1.6	90.8–86.8	89.2|1.5
Raw signal	77.2–74.0%	75.3%|1.2	68.1–64.7	66.2|1.2	89.4–86.3	87.5|1.3
QRS	77.1–75.1%	75.8%|0.8	69.6–66.8	67.9|1.0	90.9–87.5	89.6|1.3
QRS, QRS entropy, Raw signal, Raw signal entropy	76.9–74.8%	75.8%|0.8	68.4–66.2	67.4|0.9	91.5–90.1	90.9|0.5
QRS, Raw signal	76.7–74.9%	75.8%|0.6	68.6–66.4	67.7|0.9	91.7–89.7	90.5|0.7
QRS, Raw signal entropy	76.5–73.5%	75.5%|1.2	68.4–65.0	66.9|1.2	90.1–88.3	89.6|0.7
QRS entropy, Raw signal	76.5–74.7%	75.8%|0.6	68.9–65.8	67.1|1.3	90.4–89.2	89.8|0.4
Raw signal, Raw signal entropy	76.2–73.9%	75.1%|1.0	66.8–61.8	64.4|1.8	88.9–87.0	88.2|0.9
QRS entropy, Raw signal entropy	70.5–68.2%	69.3%|0.9	61.7–57.2	59.3|1.8	88.5–86.7	87.3|0.6
QRS entropy	70.0–68.0%	68.8%|0.7	60.4–58.3	59.3|0.8	87.4–86.4	87.0|0.4
Raw signal entropy	65.1–63.7%	64.3%|0.5	54.4–52.0	53.2|0.9	85.3–83.1	84.4|0.9

**Table 8:** Results for 20-class classification.

Name	Acc	Acc Avg|Std	F1	F1 Avg|Std	AUC	AUC Avg|Std
QRS, QRS entropy, Raw signal, Raw signal entropy	70.8–66.4%	67.6%|1.7	34.5–31.8	33.6|1.0	87.9–84.6	86.1|1.1
QRS, Raw signal	70.3–65.4%	67.5%|1.9	34.3–30.4	32.9|1.5	83.3–82.3	82.7|0.3
QRS, QRS entropy	70.2–66.6%	67.6%|1.5	37.0–31.7	34.1|2.0	87.0–84.7	85.7|0.9
QRS, QRS entropy, Raw signal	70.2–66.6%	68.5%|1.3	34.7–33.1	34.1|0.6	86.4–83.2	85.3|1.2
QRS, Raw signal entropy	69.7–65.3%	67.2%|1.6	36.3–31.3	33.9|2.0	86.8–84.1	85.2|1.1
QRS, QRS entropy, Raw signal entropy	69.6–66.5%	67.5%|1.3	36.3–32.7	34.5|1.4	86.2–85.0	85.7|0.5
QRS, Raw signal, Raw signal entropy	69.6–66.6%	68.2%|1.1	35.2–32.1	33.6|1.1	86.0–83.0	84.4|1.1
QRS entropy, Raw signal, Raw signal entropy	68.2–64.2%	66.2%|1.5	30.6–27.7	29.6|1.2	82.9–81.6	82.2|0.6
QRS	68.2–66.2%	67.1%|0.8	33.0–31.9	32.4|0.4	86.3–82.4	84.4|1.5
Raw signal, Raw signal entropy	66.3–63.0%	64.3%|1.2	29.8–26.9	28.0|1.1	82.1–77.6	79.5|1.8
QRS entropy, Raw signal	66.2–63.4%	65.1%|1.0	31.1–28.5	29.2|1.1	83.5–80.0	81.8|1.2
QRS entropy, Raw signal entropy	63.3–61.3%	62.0%|0.8	28.2–26.5	27.6|0.7	84.7–82.9	83.8|0.7
QRS entropy	62.3–58.8%	60.2%|1.4	25.7–22.2	23.9|1.5	82.8–80.5	82.0|0.9
Raw signal	62.0–59.9%	61.3%|0.8	26.9–24.4	25.6|0.9	76.0–73.7	75.1|1.1
Raw signal entropy	59.1–55.9%	57.2%|1.1	22.1–21.1	21.5|0.4	80.7–78.8	79.7|0.8

## Data Availability

The data presented in this study are available on request from the corresponding author.

## Nomenclature

ECG	Electrocardiogram
QRS complex	Combination of three of the graphical deflections (Q wave, R wave, and S wave) seen in a typical ECG record. It represents an electrical impulse spreading through the ventricles of the heart and indicating their depolarization
Conv1d	Layer in Deep Neural Networks that performs a convolution on a one-dimensional signal
MaxPool1d	Layer in Deep Neural Networks that performs a pooling operation by selecting the maximum value from the moving window
Fully-Connected	Layer in Deep Neural Networks that consists of neurons, each of which process the whole of the input data
Leaky ReLU	Activation function used in Deep Neural Networks
Padding	Parameter used in convolutional layers specifying the amount of zeroed samples added to the start and end of the processed signal. For example, a padding of 1 means that there is one sample of value zero artificially added at the beginning and at the end of the signal. This operation is conducted in order to mitigate activation map shrinkage due to application of convolution
Stride	Parameter used in convolutional layers specifying the shift distance between subsequent windows of convolutions. For example, a stride of 1 means that the next convolution starts right after the the beginning of the previous one, so the windows will overlap (provided that kernel size is bigger than 1)
